# Training Behavior Analysis Graduate Students to Work with an Interpreter

**DOI:** 10.1007/s40617-024-00938-w

**Published:** 2024-05-30

**Authors:** Danika J. Vazquez, Sarah A. Lechago, Morgan J. McCarville

**Affiliations:** School of Human Sciences and Humanities, Clinical Health Applied Sciences, University of Houston-Clear Lake, 2300 Bay Area Boulevard, Houston, TX 77058 USA

**Keywords:** Linguistic diversity, Hispanic, Latinx, Interpreter, ABA services

## Abstract

There has been a substantial increase in the racial and ethnic diversity of the United States population in the past 10–12 years, with the second most prevalent racial or ethnic group being Hispanic or Latino (Jensen, 2021). As a result, it is crucial that behavior analysts are prepared to serve consumers from all backgrounds, including those who do not speak English fluently. One important component for service delivery for linguistically diverse consumers is the incorporation of an interpreter. Given that few peer-reviewed articles in behavior analysis have been published regarding working with interpreters, the current study evaluated the effectiveness of Behavioral Skills Training (Fleming et al., 1996 *Journal of Organizational Behavior Management*, 16(1), 3–25) to teach behavior analysis graduate students to work with an interpreter during behavior analytic service provision with Spanish-speaking families. The results of this study show that practitioners can be trained to work with an interpreter in a relatively short amount of time. However, training with an interpreter did not affect caregiver comprehension. The results of the satisfaction surveys suggest that the interpreters noted significant improvements in the practitioners’ responding following training, whereas the caregivers did not. The participants also completed satisfaction surveys following the study and indicated positive experiences with the training.

At present, 1 in 36 children is diagnosed with autism spectrum disorder (ASD; Centers for Disease Control & Prevention, 2023). Although ASD is prevalent among all racial and ethnic groups, White children tend to be diagnosed with greater frequency and receive intervention earlier than Black, Indigenous, and people of color (BIPOC) children, which can be explained by the greater access to health care and education that white families in the United States receive (Harris et al., 2014). In particular, white children are 19% more likely to be diagnosed with ASD than Black children and 65% more likely than Latinx children (Aylward et al., 2021). Because of the delay in diagnosis, diverse families are less likely to receive early intervention services, facing obstacles due to economic stressors, language barriers, and a lack of culturally responsive care on the part of therapy-providing professionals (Aylward et al., 2021; Vega et al., 2016).

Given the numerous potential disadvantages faced by individuals with ASD from culturally diverse backgrounds, it is crucial that behavior analysts be trained to properly support these clients by providing services in a culturally sensitive manner (Frey, 2020). Social validity, the acceptability or viability of an intervention, is a cornerstone of applied behavior analysis (ABA) practice and requires culturally responsive care especially when the behavior analyst and the consumer come from different cultural backgrounds (Schwartz & Baer, 1991). The *Ethics Code for Behavior Analysts* from the Behavior Analyst Certification Board (BACB, 2020) requires behavior analysts to obtain the training and experience necessary to provide adequate services to clients from diverse backgrounds (BACB Code 1.07) and to ensure their competence by increasing their knowledge of cultural responsiveness (BACB Core Principles). Acknowledging and responding effectively to the cultural and linguistic diversity of the consumer is an important feature of providing effective and ethical ABA services.

Not only is providing interpreter services ethical, but it is also a legal requirement in many health-care and educational fields. For example, Section 300 of the Individuals with Disabilities Education Act states that agencies such as schools must take necessary action for parents to understand the proceedings during Individualized Education Program (IEP) meetings, including providing an interpreter (Individuals with Disabilities Education Act, 2004). At present, the BACB (2017) does not require any cultural competency coursework as part of graduate training or to be eligible to sit for the certification exam. However, their March 2022 newsletter announced that diversity, equity, and inclusion (DEI) content must be incorporated into behavior analytic coursework beginning in 2027 (BACB, 2022). Thus, behavior analysts need to continue evaluating methods to identify and train culturally responsive behavior analytic practices as part of an individual’s graduate training.

Previous publications that provided practitioner recommendations regarding using interpreters have been published in behavior analytic journals. Dennison et al. (2019) identified several barriers that may prevent culturally and linguistically diverse families from accessing adequate services, and ways in which ABA practitioners can help overcome these obstacles. The seven barriers include: (1) a lack of diversity in research; (2) a lack of diversity among practitioners; (3) cultural biases of the practitioner; (4) language discrepancies; (5) understanding one’s own culture; (6) practitioner understanding of socioeconomic and sociocultural stressors; and (7) a lack of understanding of multilingualism. When discussing ways to reduce language discrepancies, the authors recommend working with an interpreter, using empathy when faced with cultural conflicts, and translating documents. Although these are helpful recommendations, no experimental analysis of their effectiveness has been conducted to this point. Additional suggestions for the provision of services for culturally and linguistically diverse clients come from Fong and Tanaka (2013). They proposed the *Standards for Cultural Competence in Behavior Analysis* to provide a framework for behavior analysts to effectively deliver treatment to diverse populations. One standard states that providers should deliver information and services in the preferred language of the client or make referrals when they are incapable of doing so.

Because culturally and linguistically diverse individuals face significant barriers to receiving appropriate services, it is crucial that their practitioners be prepared to provide them with language supports to facilitate high-quality treatment. Dowdy et al. (2021) presented strategies for working with an interpreter as a board certified behavior analyst (BCBA) serving linguistically diverse clients. They described various skills to engage in before, during, and after the session and created the *Checklist for BCBA and Interpreter Service Delivery* to facilitate meetings with interpreters before and after the sessions. Before sessions, Dowdy et al. (2021) recommend that the BCBA and interpreter meet to discuss expectations for the session. The BCBA and interpreter should discuss how they will respect the client’s cultural norms as well as ask about cultural identities, practices, and traditions. They should also discuss how to address reported comments and concerns from the client.

After the session, the BCBA and interpreter should meet to discuss how their expectations were or were not met, and what they can do to improve their responding during future sessions. Next, they should review what they did to demonstrate respect for the client’s cultural practices, and what they can do to improve this moving forward. The BCBA and interpreter should review client concerns and how these will be addressed during the next session. Finally, the two should discuss how they will continue to enhance the interpretation services during the next sessions.

Dowdy et al. (2021) offered their recommendations for BCBAs when working with an interpreter during clinical sessions. The first suggestion is to use nontechnical language because interpreters are trained to deliver the original message word-for-word. Therefore, further explanation of technical terms may be needed for the caregiver to understand and for the interpreter to know how to deliver the message correctly. For example, if a BCBA mentions that they will reinforce the behavior, the interpreter may not be trained in ABA and thus not know what that word means in that context or how to properly translate it. The second recommendation is to speak in simple sentences, meaning using shorter sentences with easy-to-understand vocabulary. This skill will allow the interpreter to remember everything that was said to deliver the exact message in the consumer’s preferred language. In addition, using language-specific idioms may result in difficulty finding an equivalent in the consumer’s language.

Another recommendation is to communicate directly with the client by looking at and speaking to them rather than the interpreter. This can help build rapport and make the conversation feel more natural. For example, the BCBA should speak as though the client understands them rather than telling the interpreter, “Please tell them I said __.” The BCBA should position the interpreter next to or slightly behind the client. This will encourage the BCBA to face and speak directly to the client, and to use first person language. Finally, the BCBA should be prepared to repeat and clarify for the client and interpreter, as needed.

Using the session skills recommended by Dowdy et al. (2021), the purpose of the current study was to evaluate the effectiveness of Behavioral Skills Training (BST; i.e., instruction, modeling, rehearsal, feedback; Fleming et al., 1996) on teaching behavior analysis graduate therapists to work effectively with an interpreter during ABA service provision with Spanish-speaking caregivers. Although these checklists and recommendations can serve as a reference for BCBAs, the authors did not train anyone to use the pre- and postsession checklists nor create a checklist for use during the session. For this reason, the present study sought to evaluate a training package to teach the skills recommended by Dowdy et al. (2021) for behavior analysts working with an interpreter during sessions. The researchers aimed to determine whether these skills could be quickly taught using BST and whether the skills would influence caregiver comprehension and/or the satisfaction of the caregivers and interpreters. Thus, the study investigated the extent to which the caregivers understood the information provided by the participant and the interpreter and evaluated the acceptability of this treatment by the caregivers, interpreters, and participant therapists.

## Method

### Participants

#### Therapists

There were three participants acting as therapists who were students enrolled in a master’s program in behavior analysis. The researchers recruited participants by sending an email to students in the program. The participants had to be able to commute to campus for training. There were no age or gender requirements. Participants could not speak or understand Spanish aside from basic, conversational terms such as salutations and simple sentences. This was done to ensure that the participant’s Spanish fluency did not interfere with the interpretation.

To assess Spanish language fluency, all participants were asked to complete an online assessment using the Common European Framework of Reference for languages (CEFR). The CEFR organizes language proficiency into three broad categories including basic user, independent user, and proficient user. Within each broad category, there are two subdivisions for further differentiation, including communication using basic expressions (A1) or simple and common expressions (A2), understanding of main points and brief explanations on familiar topics (B1) or understanding of complex points and spontaneous interaction with native speakers (B2), as well as understanding a variety of long points and flexible language use (C1) or understanding of almost everything with precise communication in complex situations (C2; Council of Europe, 2022). Therapists were required to score a level A2 or lower for Spanish. Caregivers would have ideally scored a level A2 or lower for English; however, this was not a requirement as the purpose of the study centered around the graduate students’ responding. In addition, living in the United States meant it was likely that caregivers included in the study had some experience with English.

Isabela was a 24-year-old Latina who was a 2nd-year student in the behavior analysis master’s program. She received a CEFR score of A2. Isabela had no prior experience using an interpreter. Rosario was a 27-year-old Asian woman who was a 2nd-year student in the behavior analysis master’s program. She received a CEFR score of A1. Rosario had minor experience using an interpreter for the Hokkien language. Griselda was a 25-year-old Latina who was a 3rd-year student in the behavior analysis master’s program. She understood some Spanish but could not speak it and received a CEFR score of A2. Griselda had previous experience using an American Sign Language interpreter, but not as an interpreter for vocal communication.

#### Caregivers

The participants communicated with a Spanish-speaking caregiver who spoke little to no English. The caregivers in this study had immigrated from Mexico, El Salvador, and Venezuela within the last 10 years. Caregivers were recruited by contacting those who were on a waiting list to receive services through a university-based ABA center for children and adults with developmental disabilities. They were required to have a child of any age with a developmental disability, as reported by their initial questionnaires completed to obtain a spot on the center’s waitlists. There were no age or gender requirements. The caregivers had to speak Spanish as their primary language and would ideally speak/understand little to no English (i.e., basic phrases only). This was to ensure that the caregivers did not obtain the information asked in the caregiver comprehension questionnaires by understanding what the therapist said in English. The caregivers who participated in this study reported that they did not speak or understand English.

The caregivers also completed the CEFR assessment, but for English. Caregiver 1 received a 0 (unable to assess level due to lack of correct responses), Caregiver 2 received a C2, and Caregiver 3 received a 0. It is important to note that the CEFR assessment is a literacy-based assessment. Caregiver 2 reported that although she felt confident in her English reading capabilities, she was not able to speak or understand English when it was spoken to her and regularly required an interpreter for service provision. Each participant worked with a different caregiver.

#### Interpreters

In addition to the graduate therapists, graduate students who spoke both Spanish and English fluently attended sessions to facilitate communication between the therapist and the caregiver. These individuals were university students recruited via email. There were no age or gender requirements. Although these individuals were not formally trained and working as professional interpreters, they will be referred to as interpreters from this point forward for ease of understanding. It is common with service delivery for other therapists, therapist assistants, or multilingual family members to act as interpreters when a professional interpreter cannot be involved. The interpreters in this study were of Puerto Rican, Cuban, and Mexican descent. To assess Spanish language fluency, all potential interpreters also completed the CEFR assessment.

To act as an interpreter in the study, individuals needed to report previous experience speaking and understanding Spanish and score in the B2 level or higher of the CEFR assessment. This level indicated that the speaker could express themselves on a range of topics within the language, which would be important to ensure accurate interpretation. English fluency was not assessed because all interpreters grew up in the United States and attended schools where English was the primary language of instruction. Interpreter 1 received a C1, Interpreter 2 received a C1, and Interpreter 3 received a C2. Interpreters 1 and 2 had previous experience serving as interpreters during ABA sessions.

Institutional review board (IRB) approval was obtained prior to recruiting participants, including caregivers and interpreters. All individuals in the study provided informed consent following a meeting with the primary investigator to explain the nature of the study, expectations for participation, compensation, risks, and ability to withdraw at any time. There was no financial compensation for therapists for participating in the study. Therapists were taught new skills to improve their service provision. Interpreters and caregivers received a $20 gift card and caregivers were also offered the next available spot at the university-based clinic from the waitlist on which they were already listed prior to the study (i.e., participation in the study did not result in their being placed on the waitlist to begin with). This benefit was included to ensure fair compensation for caregivers for their time and travel because the majority of studies conducted with caregivers at this center focused on teaching skills to their children, but this study aimed to teach therapists instead.

### Setting and Materials

All training sessions were conducted in a designated room reserved by the first author at a university-based autism services center. Training sessions included a table and chairs, writing materials, written scenarios, caregiver questionnaires, and a behavior checklist following the first baseline phase. During rehearsal with feedback, the first author acted as the caregiver.

The first author created three categories of scenarios for caregiver meetings which included intake, developing treatment goals, and discussing the current intervention. The therapist received a new scenario before each session and was allowed to ask the primary researcher any questions related to the material. These scenarios also included information that corresponded to questions on the caregiver questionnaires. Therefore, each of the three categories included three variations such that the main idea stayed the same, but the details varied. For example, the scenarios varied in the goal of the next session and what to do before the next session. This was done to allow for multiple options when completing the *caregiver comprehension questionnaire*. The primary researcher informed the caregiver that they could answer the therapist’s questions about their own child or respond hypothetically, given that the therapist was not actually providing services to the family; however, all caregivers spoke about their actual children. Please refer to Appendix Table 5 for examples of scenarios provided to participants.

### Experimental Design

A nonconcurrent multiple baseline across participants design was used to evaluate the effectiveness of the training package. Training was introduced after a predetermined number of baseline sessions with a stable or decreasing trend. A minimum of three baseline sessions for participant 1 was required to show a clear trend, whereas five sessions for Participant 2 and seven sessions for Participant 3 were selected for verification purposes of the multiple baseline design to show at least two more sessions than the prior participant. All baseline and training sessions were 5 min in duration. This length was selected so the participants had several opportunities within the session time to engage in the target responses, not including those that only had one opportunity per session (i.e., position interpreter). Because the caregiver was not a real client, there was limited information to be discussed between parties. In addition, because these were not actual therapy sessions, caregivers did not provide in-depth information about their children in response to the therapists’ questions. Using shorter sessions also aimed to show the efficiency with which therapists could be trained to allow for application to clinical or university settings with limited time and resources.

#### Response Measurement and Response Definitions

The first dependent variable was the percentage of steps for working with an interpreter that were correctly completed by the participant. This was calculated by collecting frequency data on correct and incorrect responding during each opportunity for each of the target behaviors. Then the researcher graphed the data as a percentage by dividing the number of steps completed correctly by 5 and multiplying by 100. Because some steps had multiple opportunities throughout the session, one incorrect opportunity out of five would result in that step being considered incorrect. This made the mastery requirements more stringent.

The second dependent variable was the percentage of correct responding by the caregiver on a *caregiver comprehension questionnaire* administered to the caregiver after each session. This was calculated by dividing the number of questions answered correctly by 5, the total number of questions, then multiplying by 100. Appendices B and C display the *caregiver comprehension questionnaire*. Data were not scored on whether the participants mentioned each bullet point included on the scenarios that were provided during sessions. However, before each session they were informed of which bullet points must be mentioned (e.g., date/time of the next session) to ensure that caregivers did not emit an incorrect response on the comprehension questionnaire solely because the participant never stated the correct answer during the session. Participants never had to be reminded to state one of the required bullet points.

During all sessions, the primary researcher recorded the participant’s responding using the Countee data collection application on an iPhone. The primary researcher scored each of the five target behaviors as correct or incorrect each time there was an opportunity. The first behavior was positioning the interpreter, which was defined as the participant providing a statement to the interpreter regarding positioning next to the caregiver (e.g., please stand next to the caregiver) within 30 s of the start of the session. The second target behavior was nontechnical language, which was defined as the participant using layman’s terms when speaking while seated at the table. The researcher had a list of ABA terminology that would be considered technical language. Participants could use those words only if they provided a definition or synonym immediately after.

The third target behavior was using simple sentences, which was defined as the participant speaking for up to 7 s (i.e., actively speaking), excluding pauses or “um” or repeating the same word/phrase back-to-back (e.g., “Do you have, do you have”). This definition was determined by practicing with an interpreter to determine how much information a speaker could deliver without the interpreter forgetting some of the information when relaying it. The fourth target behavior was speaking directly to the caregiver, which was defined as the participant orienting their face and body towards the caregiver, unless the interpreter was addressing them or the participant was speaking directly to the interpreter, excluding telling them to interpret.

The last target behavior was repeating/clarifying, which was defined as the participant restating or rephrasing the word or phrase when the interpreter or caregiver expressed that they did not understand or did not hear. Some behaviors presented more opportunities for emission per session than other behaviors. For example, data collectors recorded an opportunity for “using simple sentences” every time the interpreter spoke, whereas “positioning the interpreter” only offered one opportunity for emission at the start of the session. Occasionally, skills could be scored as “N/A” if there was no opportunity. This only occurred if the interpreter or caregiver never asked for something to be repeated or clarified, or if the interpreter sat next to the caregiver prior to being instructed, which happened once for Participant 3.

## Interobserver Agreement (IOA)

The 5-min sessions were exported then converted into 10-s intervals for the purpose of calculating interobserver agreement (IOA). This was done because the Countee application requires a specific interval length to be chosen to calculate proportional IOA. The researchers selected 10-s intervals to ensure that the IOA calculation was sensitive to those skills which were scored every time the participant spoke (i.e., nontechnical language, simple sentences, and speaking directly to the caregiver). All training sessions were video-recorded and at least 30% of sessions during each phase (i.e., Baseline 1, Baseline 2, training, posttraining) with each participant were scored by secondary observers to collect IOA.

The secondary observers were trained using BST to collect data on the participant’s performance using the same Countee layout as the primary researcher. Data collectors were provided with examples of technical language that would result in an incorrect response for the “nontechnical language” target based on instances observed in regular sessions within the center. The secondary observers were considered fully trained when they had at least 90% agreement with the primary observer for one session to ensure a high level of competency. IOA data were calculated using proportional IOA in which the researchers calculated the sum of the lower frequency divided by the higher frequency for each interval then divided the result by the total number of intervals and multiplied by 100 to obtain a percentage. Across all phases for Isabela, IOA was assessed for 33% of sessions and the average percentage of agreement was 95% (range: 81%–100%). Across all phases for Rosario, IOA was assessed for 39% of sessions and the average percentage of agreement was 97% (range: 80%–100%). Across all phases for Griselda, IOA was assessed for 36% of sessions and the average percentage of agreement was 96% (range: 82%–100%).

## Procedures

Participants attended at least one and a maximum of three 30- to 60-min meetings per week, during which at least three sessions were conducted, depending on the time available. Most meetings included three or four sessions. Prior to baseline, the primary researcher obtained informed consent from the participant therapist, interpreter, and caregiver.

## Interpreter Training

The primary researcher met with the interpreter to review expectations and provide a brief training on how to serve as an interpreter depending on their previous experience, with help from the recommendations outlined in the Dowdy et al. article (2021). It is important to note that this was not formal training that a professional interpreter may go through, but rather a review of participation requirements and basic instructions. The training involved telling the interpreter to relay the information word-for-word as best as possible to each party and to wait for the participant to stop speaking before interpreting.

The interpreters were told to match the tone and emotion being displayed by the participant, because these are crucial parts of a conversation and could influence the caregiver’s satisfaction. The first author reviewed the target behaviors with the interpreter, as well. This was done to ensure that the interpreter’s behavior did not serve as a confounding variable in the study by affecting the caregiver’s comprehension (e.g., reframing the participant’s sentence from technical to nontechnical language). The interpreter was also told that the participant may occasionally speak too quickly or for too long, resulting in difficulty relaying the exact information to the caregiver. This was mentioned so interpreters did not attempt to summarize or restate this information. Interpreters were instructed to ask for clarification or to have the participant repeat the information if they did not hear or recall it so all information was accurately relayed to the caregiver. The interpreters were asked to do this at least once per session to provide the participant with an opportunity to engage in the target behavior of repeating or clarifying a statement following a question from the interpreter or caregiver. Interpreter integrity was not formally evaluated in the present study.

## Baseline

### Baseline 1

During the first baseline phase, the participant conducted a 5-min caregiver meeting. They were told that they would be meeting with a caregiver who did not speak English, so an interpreter would be provided. The researcher provided no further instructions on how to work with the interpreter. The participant then received a scenario with talking points for them to go over with the caregiver during the meeting. The researcher did not program any consequences for correct or incorrect responding. After each session, the caregiver completed the *caregiver comprehension questionnaire*. Once the participant’s responding was stable or on a decreasing trend, they moved to the second baseline phase.

### Baseline 2

The researcher included a second baseline phase with the same procedures except the participant was given a checklist with the target behaviors, excluding definitions. The checklist included the following steps: (1) Position the interpreter; (2) Nontechnical language; (3) Simple sentences; (4) Speak to caregiver; and (5) Repeat/clarify. The researchers included this phase to determine whether responding would increase with the checklist alone, or if training was required to produce correct responding. Once responding was stable or on a decreasing trend, the participant moved to BST. If a participant’s responding met the mastery criterion during this phase, they would not participate in the training portion of the study, though this did not occur in this study.

## BST

After completing the two baseline phases, the participants initiated the training portion of the study. The BST condition involved three steps including instruction, modeling, and rehearsal with feedback (Fleming et al., 1996). During the instruction section, the participants received the checklist, and the researcher explained the definitions of correct and incorrect responding for each behavior. The participants could then ask questions. During the modeling portion of training, the participants watched a video model of a caregiver meeting using an interpreter in which the model therapist engaged in each of the target behaviors. Again, the participants were invited to ask questions.

Next, the participant practiced caregiver meetings using the three different scenarios with the same interpreter, with the first author acting as the caregiver. The first author alternated between three client profiles with specific information about the child that were kept constant across participants to ensure each participant experienced the same opportunities. The primary researcher answered questions similarly to how caregivers answered during baseline (e.g., describing their child and the goals they’d like to achieve). The primary researcher provided delayed feedback at the end of each session including praise and corrective feedback. The participant was allowed to ask questions at this time as well. Once the participant engaged in 100% of the steps correctly for three consecutive sessions, they moved to the posttraining probes. Mastery was set at 100% to ensure a high level of competency prior to working with the actual caregivers again.

If a participant’s responding did not meet the mastery criterion, the first author added a visual prompt to increase responding. The visual prompt involved the first author holding up their finger once the participant had been speaking for 4 s. The researcher kept the finger up until the participant reached the 7-s limit. Once the participant’s responding increased to 100% correct across three consecutive sessions, the researcher faded the prompt by only holding the finger up for 1 s at the 4-s mark. After three consecutive sessions with 100% correct responding, the primary researcher removed the prompt entirely. The participant then needed to demonstrate 100% correct responding across three consecutive sessions before moving to the posttraining probes.

## Posttraining Probes

After completing BST, the participants met for 5-min sessions with the same Spanish-speaking caregiver they met with during baseline sessions. The participants were presented with scenarios and the checklist. The participants had been exposed to the scenarios during baseline and rehearsal with feedback; however, the purpose of the scenarios was to provide therapists with sufficient information to fill the session time and ensure the caregivers were given the information needed to complete the comprehension questionnaires. Once the participants engaged in all the target behaviors at 100% correct for two consecutive session probes, they completed the study. Had a participant engaged in less than 80% correct responding across two consecutive sessions, they would have been provided remedial training which would have involved reviewing the checklist, rewatching the video model, and practicing with the primary researcher and interpreter until their responding increased to 100% correct across two consecutive sessions. However, this was not necessary in the current study.

## Caregiver Comprehension Questionnaire

After each phase throughout the study (baseline and posttraining) in which actual caregivers participated, the caregivers were administered a *caregiver comprehension questionnaire* to determine the extent to which they could effectively respond to (i.e., understand) the information provided to them during the session by the therapist and interpreter. The caregiver completed the questionnaire using a writing utensil. Each questionnaire was presented in Spanish and included the same five questions, “What was the objective of this session?,” “What did the therapist say to do before the next session?,” “What is the date and time of the next session?,” “Where is the next session?,” and “What is the plan for the next session?” The scenarios included slight variations in correct answers to each question so the caregiver would need to understand what the therapist stated to distinguish between the potential answers for each question. The researcher included this questionnaire as a social validity measure to assess the effects of the training package on caregiver comprehension as a form of collateral behavior that would potentially be influenced by the changes in the target behaviors of the first dependent variable.

## Social Validity

The researcher provided satisfaction surveys to the caregivers and interpreters regarding their experience within the study to show whether they felt the participants demonstrated improvements after BST. They were assured that the participants would never see their responses and the purpose of the surveys was to learn about their experience within the study. They were instructed to select a number between one (i.e., strongly disagree) and five (i.e., strongly agree) to indicate their agreement with the statements related to how they felt during the study before and after the intervention. The questions corresponded to the specific target behaviors and are included in Tables 1 and 2 along with the average responses.

**Table 1:** Results of Caregiver Satisfaction Survey

Question	Average score	
	Pre-training	Posttraining
The therapist spoke in words and phrases that were easy to understand.	5	5
The therapist’s expectations for me were clear.	5	5
The therapist paid attention to me rather than the interpreter.	5	5
The therapist made me feel comfortable using an interpreter.	5	5
I felt respected and included by the therapist.	5	5

The results of the participant therapist satisfaction survey are described above, with the average score being 5/5 for each question following the termination of the study, except their level of comfort using an interpreter prior to training (average of 3).

**Table 2:** Results of Interpreter Satisfaction Survey

Question	Average score	
	Pretraining	Posttraining
I was easily able to communicate everything the therapist said when it was time to interpret.	3	5
The therapist spoke using terms and phrasing that were easy to translate (i.e., minimal slang or technical terms).	3	4.66
The therapist’s expectations of my role were clear.	3.66	5
The therapist focused their attention on the caregiver rather than on me.	3	5
The therapist made the caregiver feel comfortable using an interpreter.	3.66	4.66
I felt respected and included by the therapist.	4.66	5

The results of the interpreter satisfaction survey are described above, showing an increase in scores for each question following training.

Following the completion of the study, participant therapists also completed a satisfaction survey regarding the perceived importance of the training and their preference for it. The survey format followed that of the caregiver and interpreter surveys in terms of rating agreement. The questions are included in Table 3 along with the average responses.

**Table 3:** Results of Participant Therapist Satisfaction survey

Questions	Average Score
Learning how to work with an interpreter is an important skill for behavior analysts.	5
The provided training improved my skills for working with an interpreter.	5
The skills taught in this training are skills that ABA practitioners should use when working with an interpreter.	5
The training I received was efficient	5
I felt comfortable using an interpreter PRIOR to this training.	3
I felt comfortable using an interpreter AFTER this training.	5
I would recommend this training to other behavior analysis graduate students.	5
I would recommend this training to practitioners working in behavior analytic clinics.	5

The results of the participant therapist satisfaction survey are described above, with the average score being 5/5 for each question following the termination of the study, except their level of comfort using an interpreter prior to training (average of 3).

## Results

**Fig. 1:**
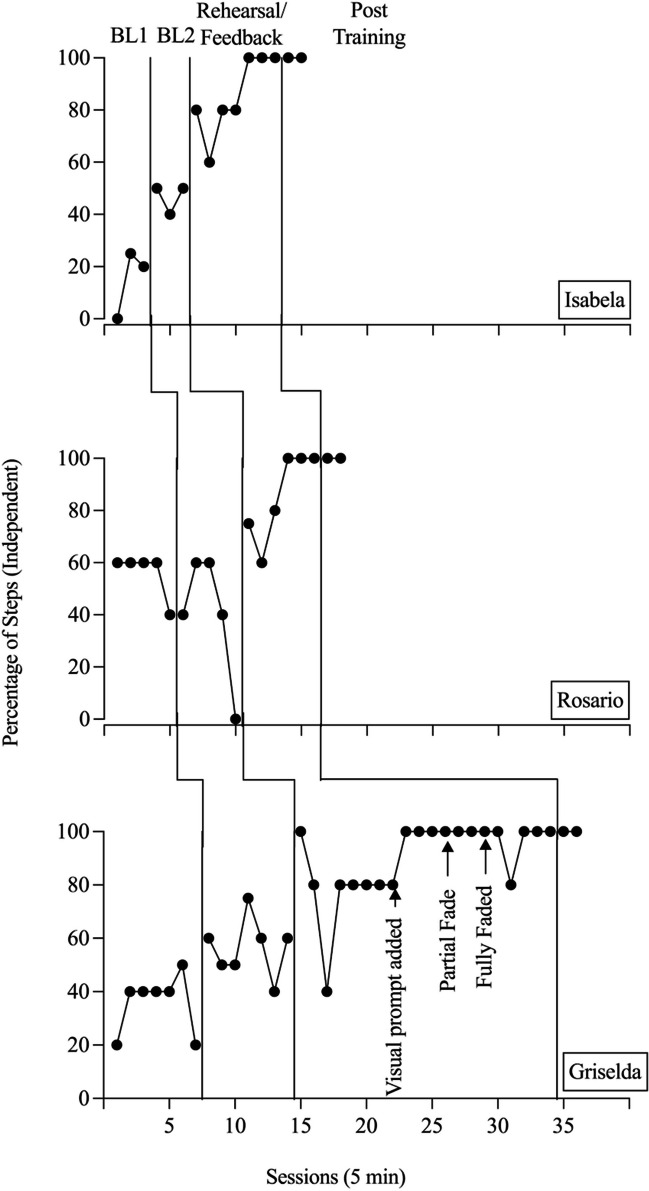
Correct Implementation of Interpreter Interaction Skills. *Note.* Results of behavior skills training for Isabela (top), Rosario (middle), and Griselda (bottom).

Figure 1 represents the participant therapists’ responding throughout all phases of the experiment. Caregiver comprehension data were collected during baseline and posttraining. No caregiver comprehension data were collected during rehearsal/feedback because the primary researcher was acting as the caregiver, spoke fluent English and Spanish, and was familiar with the scenarios. During the first baseline phase, all participants engaged in 60% or fewer target behaviors correctly. Once the researcher provided the checklist in the second baseline phase, responding increased slightly for two of the three participants. However, none of the participants completed all target behaviors correctly with the checklist alone. For Rosario, correct responding decreased with the provision of the checklist compared to the first baseline phase.

Isabela’s responding is represented on the top panel of Figure 1. During Baseline 1, her responding was low and variable. During Baseline 2, her responding was moderate with a stable trend, indicating that the checklist, alone, was helpful in improving performance. Once BST was initiated, her responding was high and on an increasing trend, meeting the mastery criterion after seven role play sessions (session 13). After role play, she then conducted two posttraining probe sessions with the caregiver. Her responding remained stable at 100%, showing generalization of the skills. The first caregiver’s comprehension was high during Baselines 1 and 2, though it decreased with each session, ranging from 60% to 100% correct responding. During posttraining, the caregiver’s comprehension was also on a decreasing trend, also ranging from 60% to 100% correct responding.

Rosario’s responding is represented in the middle panel of Figure 1. During Baselines 1 and 2, her responding was moderate with a decreasing trend. During BST, her responding was high with an increasing trend. Her responding met the mastery criterion after six role play sessions (Session 16). Following role play, she participated in two posttraining probe sessions with the caregiver. Her responding remained stable at 100%, demonstrating generalization. During both baseline phases, the second caregiver’s responding was moderate and variable, ranging from 20% to 80% correct responding. During posttraining, the caregiver’s responding was moderate with an increasing trend, ranging from 40% to 80% correct responding.

Griselda’s responding is represented in the bottom panel of Figure 1. During Baseline 1, her responding was low and variable. During Baseline 2, her responding was moderate and variable. At the start of BST, her responding increased to 100% correct. However, there was a decreasing trend across the next two sessions. The researcher continued to use the same procedures as those used with the first two participants until responding remained stable at 80% across four consecutive sessions, showing that additional assistance was necessary. The researchers introduced a visual prompt during Session 22. Her responding increased to 100% correct across three consecutive sessions after Session 25. The researchers fully faded the prompt by Session 29. Griselda’s responding met the mastery criterion in Session 34. She then moved to the posttraining probes with the caregiver. Her responding remained stable at 100%, demonstrating generalization. During both baseline phases, the third caregiver’s responding was high and variable, ranging from 60% to 100% correct responding. During posttraining, her correct responding was stable at 80% correct responding.

To determine which skills resulted in the greatest percentage of errors, the researchers conducted an error analysis. This was done by calculating the percentage of opportunities in which an incorrect response was made for each skill during both baseline phases. The purpose of this analysis was to assist practitioners and future researchers in deciding which skills may require extra training or assistance. Results of the error analysis are displayed in Table 4. During Baselines 1 and 2 respectively, participants emitted an incorrect response for the skill of positioning the interpreter 84.3% and 66.7% of opportunities, speaking in simple sentences 56% and 39.7% of opportunities, speaking directly to the caregiver 17.7% and 14.3% of opportunities, using nontechnical language 6% and 3% of opportunities, and repeating/clarifying 36% and 9.3% of opportunities. The most commonly emitted error across participants during BST occurred with speaking in simple sentences.

**Table 4:** Results of Error Analysis

Skill of Error	Baseline 1 Average Opportunities Incorrect	Baseline 2 Average Opportunities Incorrect	Total Average Opportunities Incorrect
Position interpreter	84.3%	66.7%	75.5%
Speak in simple sentences	56%	39.7%	48%
Speak directly to caregiver	17.7%	14.3%	16%
Use nontechnical language	6%	3%	4.5%
Repeat/clarify	36%	9.3%	22.7%

The results of the error analysis are described above, with the most incorrect opportunities occurring for “position interpreter” and “speak in simple sentences” across both baseline phases. There was a decrease in errors across all skills in Baseline 2.

All caregivers, interpreters, and participant therapists in the study returned the satisfaction surveys. The results of the caregiver satisfaction surveys showed that the caregivers strongly agreed with all statements before and after intervention. However, the interpreter satisfaction surveys represent improvements following intervention. In particular, the interpreters noted the highest improvements with the ease of communicating information (Question 1), the terminology used (Question 3), and the participant attending to the caregiver (Question 4). The interpreters’ average scores are displayed in Table 2. The results of the therapist surveys indicated that they perceived the training of these skills to be significant and important, increased their self-confidence for working with interpreters (average score of 3 prior to training and 5 after training), that they highly approved the training, and they reported a high likelihood of recommending the training for others. The participant therapists’ average scores are displayed in Table 3.

## Discussion

There is a growing need within the field of ABA to provide culturally responsive care for families. However, there is still a dearth of experimental studies in ABA on diversity, equity, and inclusion in behavior analytic service provision. Similar to the present study, Gatzunis et al. (2023) trained behavior analysis students skills for working with diverse families including functional assessment interviewing, cultural responsiveness, and compassionate care. Within the realm of cultural responsiveness, participants were taught to identify clients’ preferred language; arrange for an interpreter if needed; conduct an analysis of cultural identity; refrain from using technical jargon, idioms, and offensive statements; ask clarifying questions; refrain from making assumptions about values; and identify family roles. Participants showed mastery across the skills taught and the training was highly efficient, taking an average of 4.5 hr to complete. One limitation noted in the study was the lack of practice opportunities to interview actual caregivers. The skills were also taught via Zoom without a test for generalization to in-person interviewing.

The present study also taught graduate students in behavior analysis skills related to cultural responsiveness, particularly those involved in working with linguistically diverse families and an interpreter. This study also provides evidence for the efficacy of a culturally responsive training package for working with interpreters that includes a structured checklist with operational definitions for use during training. This training can help prepare practitioners to work more effectively and sensitively with an interpreter in the context of service provision and can be conducted in a short period of time using procedures familiar to most behavior analysts. Isabela and Rosario’s responding met the mastery criterion after six to seven role play sessions, representing an average of 33 min of role play. The instruction and modeling portion of the training package took an average of 15 min to complete. Thus, training a practitioner to work with an interpreter can be completed in less than 1 hr. This is important to note because of the time and resource constraints common to ABA clinics.

Whereas Gatzunis et al. (2023) used BST to train participants via Zoom, this study was conducted entirely in person, showing that BST for skills related to cultural responsiveness can be effective for in-person sessions, as well. Unlike Gatzunis et al. (2023), the present study emphasized generalization by not only using BST to teach the skills during role-play, but also testing the skills with real caregivers.

The researchers included Baseline 2 to determine whether the checklist alone would result in correct responding. Participants could only see the specific target behaviors, not the response definitions. The results of this study show that providing the checklist without explicit training is not sufficient to produce correct responding. Previous self-instruction research has indicated that written instructions alone can be effective in improving responding (Graff & Karsten, 2012). It is possible that the participants’ responding may have been higher had they also had access to the response definitions during baseline 2. Future research should evaluate this. Further, clinics should ensure that BST is used to train employees to work with interpreters. If there are no staff members to act as a caregiver during training, significant supervision and feedback should be provided during interactions with the caregiver to ensure all skills are correctly implemented.

The first two participants’ responding met mastery with BST alone whereas Griselda required an additional visual prompt. All participants had extensive experience working in the clinic and had prior interactions with the interpreters in English, as they were also graduate students. Griselda noted that she often speaks a lot during conversations, so it did not seem natural to her to speak in such short phrases. Therefore, the visual prompt regarding time spoken was added. This prompt was effective at reducing the amount of time Griselda spoke prior to allowing the interpreter to speak. This prompt could be considered for trainees who experience similar challenges.

The error analysis also served to provide practitioners and future researchers with considerations for altering the training to focus more on skills that resulted in the most errors, positioning the interpreter and speaking in simple sentences in this case. Though the inclusion of the checklist in Baseline 2 did not produce a large increase in responding for any participants, they all emitted fewer incorrect responses during this phase, suggesting that the checklist alone can help reduce errors.

## Limitations and Future Research

The caregiver social validity measures suggest a few limitations of this study. First, the caregiver comprehension questionnaires did not show a correspondence between the participants’ correct responding and caregiver comprehension of the material. It is possible that the short duration of the sessions and simple questions made it likely that the caregivers would respond correctly. Future research could use open-ended questions rather than multiple choice questions to see if this influences comprehension. In addition, the role play style of the study may have contributed to high levels of comprehension in the caregivers. For the first caregiver, responding decreased during each meeting, indicating that her responding was highest during the first session of the day. This could suggest a decrease in motivation throughout research sessions. However, her level of comprehension remained the same at 80% throughout the study.

For the second caregiver, responding was lower than that of the other caregivers, even though she scored very high on the CEFR language assessment. The second caregiver was still allowed to participate even with a high CEFR score because the main goal of the study was to train graduate students and she reported strong difficulties with understanding spoken English. This caregiver did not have childcare and brought her toddler to the sessions. This may have created a distraction during sessions that prevented her from obtaining all the necessary information during the session. Future research should adjust the session length and modify the comprehension questions to determine if there is a correlation between caregiver comprehension and participant responding. Future studies could offer childcare to reduce distractions during the sessions and ensure that this is not a barrier to attending sessions. It is noteworthy, however, that her comprehension was on an upward trend during posttraining probes. Another method for future research to consider for caregiver comprehension would be related to questions asked by caregivers during the session. Though a time to check for questions from caregivers was not explicitly included in the scenarios provided, the therapists consistently asked the caregivers if they had any questions before terminating the interaction.

The second social validity measure was the satisfaction survey. All caregivers provided perfect scores before and after intervention, which could suggest that the participants still managed to develop good rapport with the caregivers, and the interactions were sensitive and pleasant absent the intervention. However, there are other possible explanations for these results. As part of their compensation for participation in this study, caregivers were moved up on one of the clinic waitlists they were already on and given an opportunity to receive the next available appointment for their child at a university-based center for children and adults with developmental disabilities. Therefore, it is possible that the caregivers did not want to appear ungrateful for the opportunity by providing low scores.

Another possibility is that the language barrier prevented the caregivers from recognizing the deficits in responding by the participants. There could also be cultural aspects to these ratings. For people in some cultures, it may be considered rude or inappropriate to negatively evaluate the performance of a service provider, as hypothesized by the researchers. Ways in which caregivers of different cultures provide feedback to service providers could be an area of future research to gain more insight in this area.

Moreover, the interpreter acted as the middle person to bridge communication between the caregiver and participant and received brief training before the study to ensure they understood the target behaviors and expectations to not interfere with the caregiver’s comprehension. Thus, the interpreter’s brief training may have facilitated greater understanding than would have existed had the interpreter not received prior instruction. However, the interpreters’ integrity was not evaluated during this study.

In many cases, a therapist, therapist assistant, or multilingual family member will act as the interpreter during caregiver meetings and receive no training on how to interpret effectively. Previous research has shown that having a family member serve as an interpreter often made it less likely that the information would be interpreted literally, objectively, and without emotional connection to the patient (Hadziabdic et al., 2014). Professional interpreters have been found to be more effective in reducing communication barriers than family members, with strains often arising between family members such as when interpreting difficult information like a life-threatening diagnosis (Hadziabdic et al., 2014). Future research should evaluate the role of the interpreter’s training level and treatment integrity and how these variables influence caregiver comprehension and satisfaction.

Additional socially valid aspects were evaluated in this study with regard to cultural implications. For example, caregiver comprehension questionnaires and surveys were provided in Spanish to account for their preferred language. The primary researcher or interpreter greeted the caregiver and walked them in and out of the clinic for each meeting so they could discuss in their preferred language anything outside of the topics discussed during sessions (e.g., changing the meeting day/time, number of remaining sessions). The second caregiver was also permitted to bring her child to the clinic so childcare would not serve as a barrier to attending the meetings. Future studies could incorporate more cultural measures within the satisfaction survey to measure the participants’ perceived cultural responsiveness.

Although the caregiver satisfaction surveys did not reflect changes following training due to ceiling effects, the interpreter satisfaction surveys did. This suggests that the measures selected were important for the interpreter to work effectively with the caregiver. This is valuable information because it could contribute to building rapport between the interpreter and practitioner resulting in an increase of the interpreter’s job satisfaction. The field of ABA has historically not focused on interdisciplinary collaborations, and it behooves us to contribute to the experimental literature on ways to improve respectful and positive professional teamwork and collaboration with other professionals. Unfortunately, most behavior analysts have received little to no professional development related to working in interdisciplinary teams (Slim & Reuter-Yuill, 2021). Cultural humility is not only critical to working with clients from diverse backgrounds, but also for collaborating with professionals outside of the field to strengthen the impact of services through humble behaviorism (Kirby et al., 2022).

Learning to work effectively with other disciplines as part of a clinical team serves DEI as it contributes to the diversification of knowledge and intellectual contributions. It also increases the equity of the consumer’s experience, recognizing that they will most likely require more than just behavior analytic services to reach their goals (most especially linguistically diverse families). With respect to inclusion, working more sensitively and effectively with other disciplines increases inclusion of other perspectives to service provision, potentially increases the likelihood of inclusion of behavior analysts to collaborate with others if they find it a positive working experience to do so, and importantly increases the inclusion of the consumer in their own care. A significant goal is to ensure full comprehension and positive attitudes on the part of the consumer about working with behavior analysts so they can contribute meaningfully and fully to their own and their family’s care. Working effectively with an interpreter is critical to this cause when working with linguistically diverse families.

This study contributes to the small body of experimental research on serving linguistically diverse families in ABA, and offers fruitful avenues for future research in this area. Future research should evaluate the effects of this training during actual service provision rather than short caregiver meetings. Further, evaluation of these procedures within a group training context may be beneficial in determining more time-efficient ways to train staff. Overall, this study provides support for using BST in conjunction with the checklist to train behavior analytic staff and graduate students in working with interpreters to provide services for linguistically diverse families. By carefully addressing the language barriers between staff and families through the correct use of an interpreter, behavior analysts can serve more families and develop strong rapport that can help with treatment adherence and procedural fidelity.

## Data Availability

All data generated or analyzed during this study are included in this published article.

## Appendix A

**Table 5:** Example scenarios

Intake scenario 1	Goals scenario 4	Intervention scenario 7
Introductions	Greetings	Greetings
Plan: Learn about child	Plan: Identify goals	Plan: Talk about treatment plan
Ask caregiver about their child	Ask caregiver what they want their child to learn	Discuss intervention plan
Next meeting: Mon 1 PM at their house	Next meeting: Thurs 1 PM at their house	Next meeting: Mon 1 PM at clinic
Before next session: Make list of possible goals	Before next session: Take behavior notes	Before next session: Take behavior notes
Next meeting: Identify goals	Next meeting: Talk about treatment plan	Next meeting: Talk about treatment plan

Examples of scenarios provided to participants before sessions

see Table 5

## Appendix B Caregiver comprehension questionnaire (English)

*Note.* Caregiver comprehension questionnaire shown in English. Copies were provided in Spanish following each session

## Appendix C Caregiver comprehension questionnaire (Spanish)

*Note.* Caregiver comprehension questionnaire shown in Spanish as provided to caregivers.
